# Case report: Temozolomide induced hypermutation indicates an unfavorable response to immunotherapy in patient with gliomas

**DOI:** 10.3389/fimmu.2024.1369972

**Published:** 2024-04-04

**Authors:** Jiapeng Liu, Shuli Hu, Haihui Jiang, Yong Cui

**Affiliations:** ^1^ Department of Neurosurgery, Peking University Third Hospital, Peking University, Beijing, China; ^2^ Department of Neurosurgery, Beijing Tiantan Hospital, Capital Medical University, Beijing, China; ^3^ National Clinical Research Center for Neurological Diseases, Center of Brain Tumor, Beijing Institute for Brain Disorders and Beijing Key Laboratory of Brain Tumor, Beijing, China

**Keywords:** immunotherapy, immunologic response, temozolomide, hypermutation, glioma

## Abstract

**Background:**

Temozolomide (TMZ) is a key component in the treatment of gliomas. Hypermutation induced by TMZ can be encountered in routine clinical practice, and its significance is progressively gaining recognition. However, the relationship between TMZ-induced hypermutation and the immunologic response remains controversial.

**Case presentation:**

We present the case of a 38-year-old male patient who underwent five surgeries for glioma. Initially diagnosed with IDH-mutant astrocytoma (WHO grade 2) during the first two surgeries, the disease progressed to grade 4 in subsequent interventions. Prior to the fourth surgery, the patient received 3 cycles of standard TMZ chemotherapy and 9 cycles of dose-dense TMZ regimens. Genomic and immunologic analyses of the tumor tissue obtained during the fourth surgery revealed a relatively favorable immune microenvironment, as indicated by an immunophenoscore of 5, suggesting potential benefits from immunotherapy. Consequently, the patient underwent low-dose irradiation combined with immunoadjuvant treatment. After completing 4 cycles of immunotherapy, the tumor significantly shrank, resulting in a partial response. However, after a 6-month duration of response, the patient experienced disease progression. Subsequent analysis of the tumor tissue obtained during the fifth surgery revealed the occurrence of hypermutation, with mutation signature analysis attributing TMZ treatment as the primary cause. Unfortunately, the patient succumbed shortly thereafter, with a survival period of 126 months.

**Conclusion:**

Patients subjected to a prolonged regimen of TMZ treatment may exhibit heightened vulnerability to hypermutation. This hypermutation induced by TMZ holds the potential to function as an indicator associated with unfavorable response to immunotherapy in gliomas.

## Introduction

Gliomas, the most common malignancies of the central nervous system, arise from glial cells and can be categorized into adult and pediatric subgroups according to the fifth edition of the World Health Organization (WHO) classification system (1, 2). The molecular characteristics of gliomas play a crucial role in their classification and grading. In adult diffuse gliomas, patients can be stratified into three subtypes with WHO grade from 1 to 4 based on the presence of isocitrate dehydrogenase 1 (IDH1) mutation and 1p/19q co-deletion (2). Low-grade gliomas (LGG), classified as grades 1 and 2, typically exhibit an indolent clinical course but are susceptible to frequent relapses (2). Upon recurrence, LGG may progress to grade 3 or grade 4 variants, characterized by increased malignancy and a less favorable prognosis (3). Surgical resection serves as the primary therapeutic approach for LGG patients, aiming to alleviate clinical symptoms and extend survival (4). However, due to the infiltrative growth pattern of gliomas, gross total resection of LGG is often unachievable, underscoring the importance of postoperative adjuvant therapy (5). In 2005, Stupp et al. conducted a pivotal multicenter clinical trial, demonstrating that postoperative concurrent radiochemotherapy combined with adjuvant temozolomide (TMZ) chemotherapy significantly improves the survival of glioblastoma patients (6). This seminal study solidified the role of TMZ in the treatment paradigm for gliomas.

TMZ, an alkylating agent capable of penetrating the blood-brain barrier, is currently the predominant adjuvant chemotherapy drug for glioma patients (7, 8). In the case of LGG, the prolonged administration of TMZ has been linked to the repetitive selection and elimination of tumor cells, potentially triggering tumor mutations and an escalation in malignancy (9). This phenomenon, known as TMZ-induced hypermutation, has gained significant attention in recent years, highlighting the substantial risk associated with the extended use of TMZ in the malignant transformation of LGG (10, 11). It is widely recognized that hypermutated tumors often exhibit limited responsiveness to conventional treatment modalities (11, 12). Immunotherapy, emerging as a promising approach in cancer therapy, holds potential for the management of hypermutated gliomas. The combination of immunotherapy and radiation has become a focal point of research, particularly following the recognition of the abscopal effect (13, 14). Despite these advancements, numerous clinical trials investigating immunotherapy for gliomas have encountered challenges (15–17). Therefore, the careful selection of suitable patients for immunotherapy is of critical importance and warrants urgent consideration.

Previous studies have indicated that tumor mutation burden (TMB) is a potential biomarker for predicting the efficacy of immunotherapy (18, 19). A higher TMB often implies more tumor neoantigens, correlating with a higher objective response rate (20). Consequently, physicians frequently utilize the TMB status to guide decisions regarding the suitability of immunotherapy for patients with tumors. However, recent research suggests that no direct correlation between the immunogenicity of new antigens, TMB, and the efficacy of immunotherapy has been established (21). Notably, an even reverse relationship between the immune response and TMB has been observed in several malignancies (9, 22, 23). All the evidence indicates that TMB alone may not be a robust biomarker for predicting the efficacy of tumor immunotherapy.

In this study, we present the case of a glioma patient who experienced TMZ-induced hypermutation and underwent immunotherapy. A thorough analysis and review of the patient’s genetic phenotype, tumor microenvironment, and the immunotherapy procedure were conducted. These findings contribute theoretical insights into understanding the relationship between tumor hypermutation and the efficacy of immunotherapy.

## Case presentation

### Clinical procedure

This is a 38-year-old male patient presented at the hospital on January 10, 2009, with a chief complaint of recurrent episodes of unconsciousness persisting for one month. Physical examination upon admission showed negative findings, with a Karnofsky Performance Scale (KPS) score of 90. Preoperative magnetic resonance (MR) images demonstrated the presence of a space-occupying lesion in the right frontal lobe, suggestive of a probable glioma. Consequently, the patient underwent surgical removal of the lesion, achieving gross total resection. Subsequent histopathological analysis confirmed the tumor to be an IDH-mutant astrocytoma, classified as WHO grade 2. Postoperatively, neither radiotherapy nor chemotherapy was administered, and the patient underwent close surveillance with periodic follow-up evaluations, which did not reveal any significant radiological changes.

In September 2015, a subsequent MR scan revealed abnormal signals in the vicinity of the original surgical cavity, indicating a high probability of tumor recurrence. Magnetic resonance spectroscopy analysis corroborated this finding by demonstrating an elevation in choline (Cho) levels and a reduction in N-acetylaspartate (NAA) peaks. Therefore, the patient underwent a secondary surgical procedure, during which a subtotal resection of the tumor was performed. Postoperative histopathological analysis confirmed the recurrence of astrocytic glioma, with molecular profiling revealing an IDH1 mutation, absence of 1p/19q loss, and MGMT promoter methylation at a level of 10%. Subsequently, the patient received adjuvant radiotherapy (54Gy/27 fractions) and underwent routine follow-up assessments.

In March 2017, MR revealed the presence of enhancing lesions within the surgical area, suggesting a potential recurrence of the tumor. Subsequently, the patient underwent a three-cycle course of TMZ chemotherapy, administered at a dose of 150-200mg/m^2^/day, orally for 5 days on with 23 days off. Unfortunately, follow-up MR scans indicated persistent tumor growth and inadequate treatment response. As a result, the patient was initiated on bevacizumab (BEV) therapy (10 mg/kg every 2 weeks), which led to notable symptom relief and tumor control. In October 2017, the patient experienced recurrent seizures, prompting further radiologic examinations that confirmed tumor progression. Despite repeated administration of BEV treatment, the tumor exhibited uncontrolled growth. In December 2017, the patient underwent a third surgical intervention, during which a partial resection of the tumor was achieved. Subsequent histopathological analysis confirmed the diagnosis of astrocytoma at WHO grade 4. Molecular profiling revealed the presence of an IDH1 mutation, no loss of the chromosome 1p/19q, MGMT promoter methylation at a level of 12%, and a Ki-67 proliferation index of approximately 80%. Following the surgery, the patient received dose-dense TMZ chemotherapy (100 mg/m^2^/day on a 28-day cycle, orally for 7 days on with 7 days off), in combination with immunotherapy which included intracranial and systemic immunoadjuvants. Specifically, the intracranial immunoadjuvant utilized in this study was polyinosinic-polycytidylic acid (poly I:C). It was administered via infusion into either the surgical cavity or ventricle, at a dosage of 1-2mg per injection, once daily (qd), for a total of 5 injections. The first three injections were administered concomitantly with a radiation dose of 2.0 Gy/fraction. The systemic immunoadjuvants employed in this study included poly I:C (administered intramuscularly at a dosage of 50 mg/kg per injection, every other day, for 7 injections) and granulocyte-macrophage colony-stimulating factor (GM-CSF) (administered subcutaneously at a dosage of 125 mg/m2 per injection, every other day, for 7 injections) (24). This comprehensive treatment approach resulted in the stabilization of the tumor.

In October 2018, the patient’s symptoms worsened, accompanied by radiological evidence of tumor progression. Chemotherapy and immunotherapy were discontinued, and BEV treatment was initiated. However, the effectiveness of BEV in controlling tumor growth was limited. Subsequently, a fourth surgical procedure was performed, achieving subtotal tumor resection. Postoperative pathological examination confirmed the diagnosis of astrocytoma at WHO grade 4, characterized by an IDH1 mutation, absence of 1p/19q chromosomal loss, MGMT promoter methylation at a level of 12%, and a Ki-67 proliferation index of approximately 90%. Following the surgery, immunotherapy was reattempted. By December 2018, the patient’s condition stabilized, and the immunotherapy was continued. Follow-up MR scans indicated partial tumor relief. By December 2018, the patient’s condition stabilized, and the current treatment regimen was continued. Subsequent MR scans demonstrated the tumor has achieved partial remission.

In May 2019, the patient’s condition deteriorated significantly, characterized by extensive tumor recurrence. Thus, the administration of immunotherapy was ceased, and an Ommaya reservoir was surgically implanted within the tumor site to facilitate intracranial chemotherapy drug delivery and intermittent cerebrospinal fluid drainage, aiming to alleviate symptoms. Concurrently, a biopsy was performed, confirming the pathological diagnosis as astrocytoma (WHO grade 4). However, the benefit of intratumoral chemotherapy was found to be limited, and the growth of the tumor proved challenging to control. Unfortunately, the patient succumbed to the disease on August 5, 2019.

**Figure 1:**
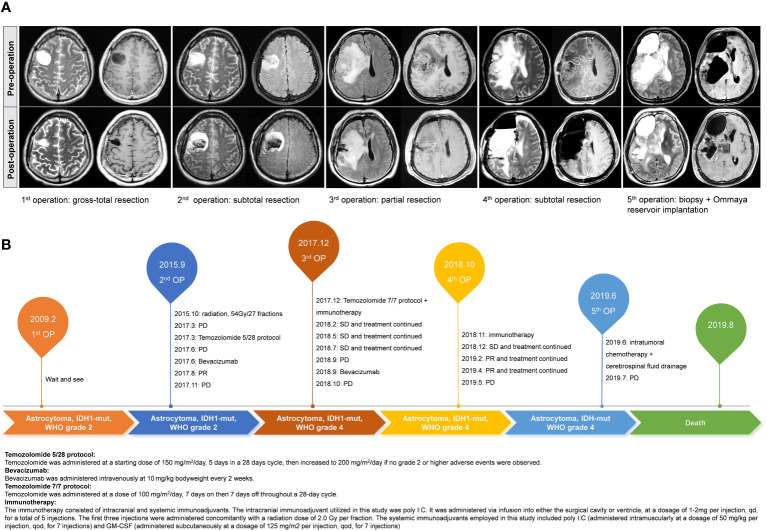
The illustrated pre- and post-operation MR images of this case during the five times of surgery **(A)**. Treatment flowchart outlining the patient’s therapeutic journey **(B)**.

The radiological images of the patient’s pre- and post-operative condition were presented in **Figure 1A**, while a detailed treatment flowchart was depicted in **Figure 1B**.

### Genomic and immunologic analysis

**Figure 2:**
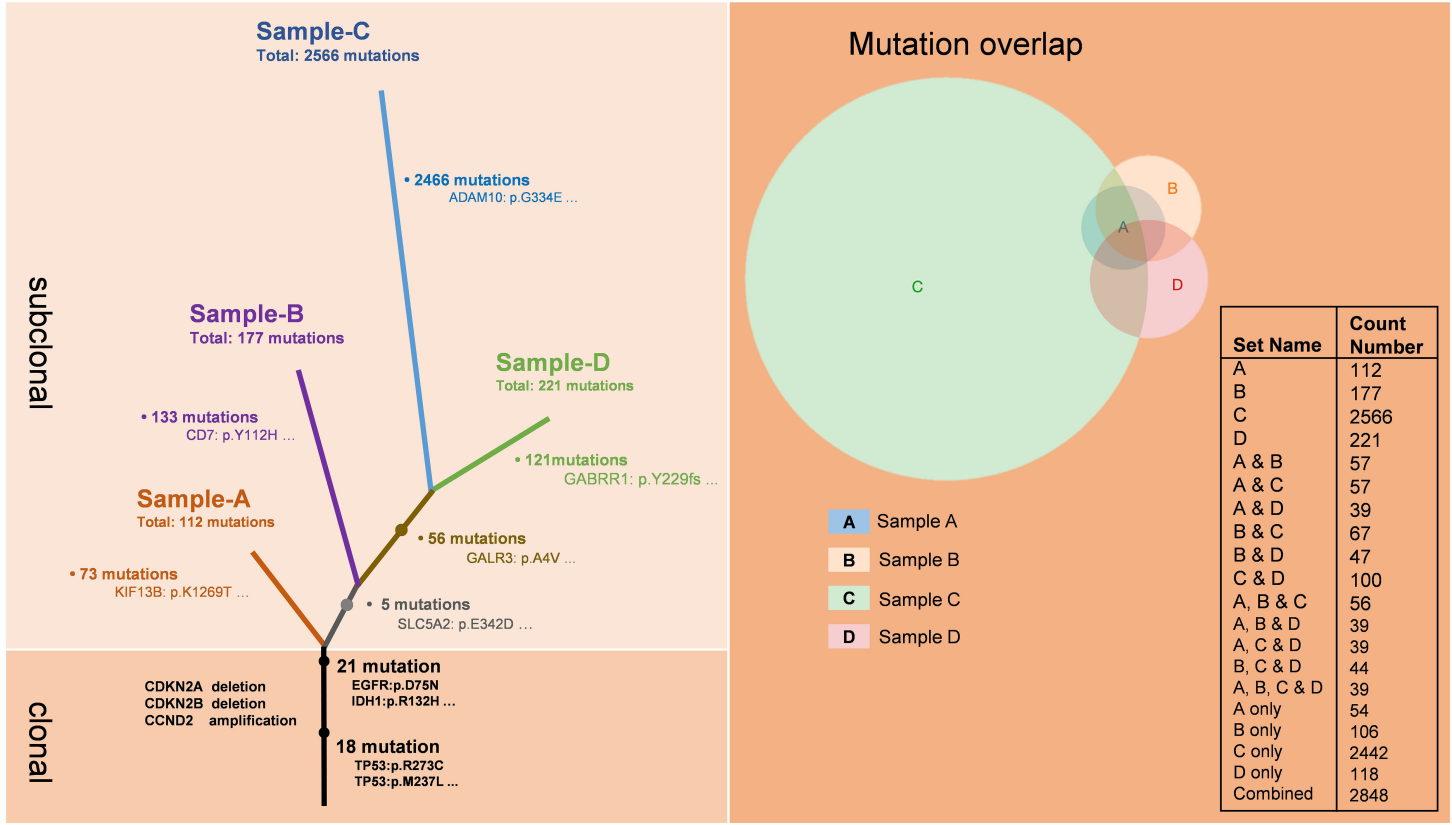
Genomic evolution of the tumor cells during recurrences.

To investigate the alterations in the tumor microenvironment pre- and post-immunotherapy, we obtained the third, fourth, and fifth tumor samples from the patient, as well as the fifth intraoperative cerebrospinal fluid sample, and conducted whole exome sequencing on these samples (**Figure 2**). The results demonstrated that all four samples shared 39 mutations from a common ancestral clone. These mutations included driver gene mutations such as TP53, EGFR, and IDH1, as well as losses of CDKN2A and CDKN2B, and amplification of CCND2. Sample A showed 73 novel mutations, sample B demonstrated 113 newly identified mutations, while sample C showcased a considerable array of fresh mutations (2466), encompassing alterations in key driver genes like APC, ERBB2, and CHEK, as well as numerous mutations related to DNA mismatch repair (MMR), such as PMS2, MSH3, POLE, ATR, and FANCA. Sample D showed 121 new mutations. Additionally, samples C and D shared 56 common mutations.

**Figure 3:**
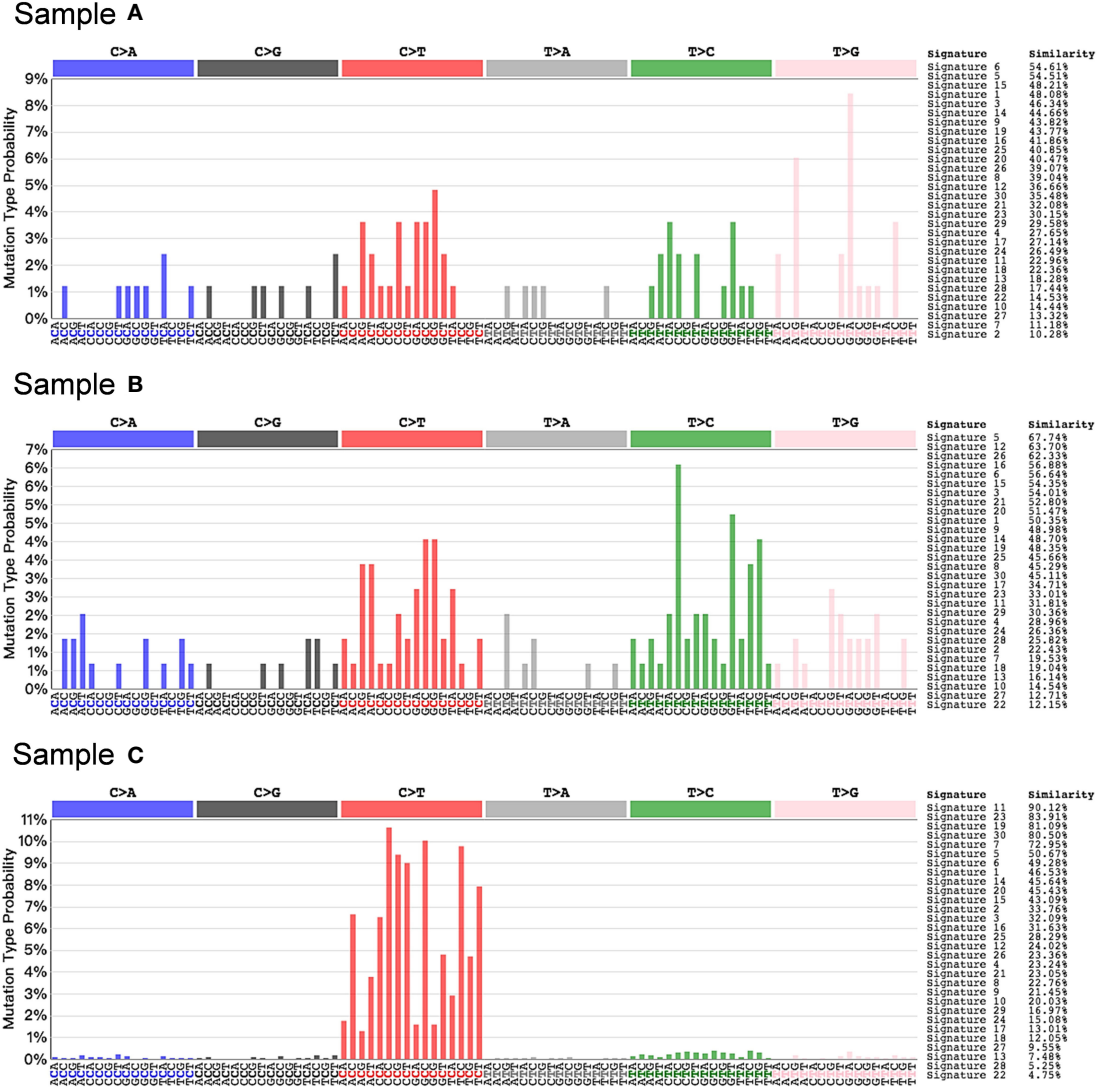
Somatic mutation signature in post-TMZ treatment tumors. Signature 11 that exhibits a strong transcriptional strand-bias for C>T substitutions was found in sample C, suggesting a mutational pattern associated with alkylating agents.

Concurrently, we performed RNA transcriptome sequencing on the third, fourth, and fifth tumor samples obtained from the patient (**Figure 3**). The results demonstrated that samples A and B exhibited no significant mutation characteristics. In contrast, sample C displayed mutation features consistent with Signature 11, indicative of a mutation pattern resembling alkylating agents (25, 26). This finding reasonably suggests that the administration of the alkylating agent TMZ likely contributed to the development of hypermutations in the tumor, thereby promoting increased malignancy.

**Figure 4:**
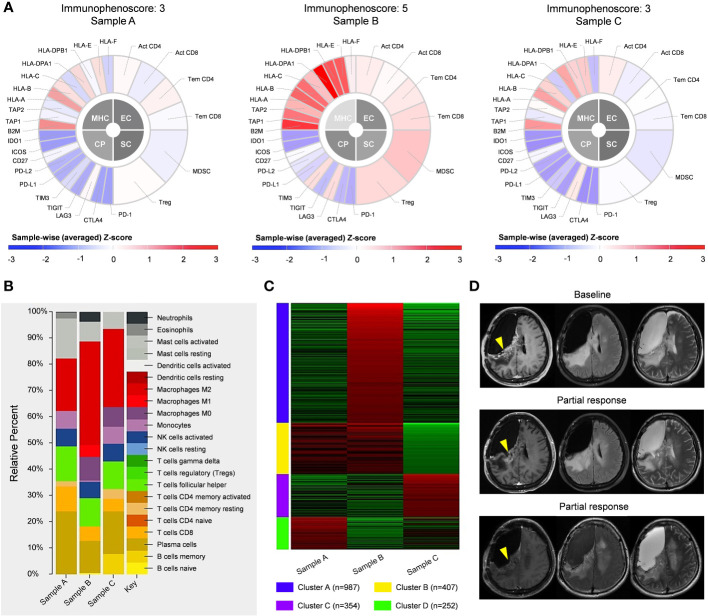
Immunologic analysis of post-operative tumors, including comparisons of immunophenoscore **(A)**, CIBERSORT results **(B)**, and enriched pathways **(C)** between different tumor samples. **(D)** Following the fourth operation, the patient received 4 cycles of immunotherapy. with partial response observed in the enhanced lesion in the right frontal-parietal lobe during follow-up.

To assess the immune microenvironment of the tumor samples, we conducted a quantification scoring system called immunophenoscore (27), which incorporates MHC molecules, immunomodulators, effector cells, and suppressor cells (**Figure 4A**). The findings revealed that the immunophenoscore for sample A, B, and C was determined as 3, 5, and 3, respectively. The CIBERSORT analysis of these samples indicated that in sample A, M2 macrophages were predominantly present. Following treatment, tumor cell death resulted in the release of a substantial amount of tumor antigens, leading to heightened immune response in sample B, as evidenced by a significant increase in the proportion of M1 macrophages (**Figure 4B**). Subsequently, in sample C, the tumor cells underwent hypermutation, rapidly deteriorating the tumor and causing the disappearance of M1 macrophages in the immune microenvironment, with M2 macrophages regaining predominance, indicative of immunologic treatment resistance. Enrichment analysis demonstrated that sample B was primarily characterized by the immune-related signaling pathway Cluster A, suggesting a favorable tumor immune response. Conversely, sample C was primarily characterized by Cluster C of the DNA damage repair-related signaling pathway, which may be attributed to TMZ-induced hypermutation (**Figure 4C**, **Supplementary Table 1**). The relatively favorable immune microenvironment observed in sample B has been validated by the sustained tumor remission following immunotherapy administration (**Figure 4D**).

## Discussion

The long-term administration of TMZ has been conclusively associated with the malignant transformation of LGG, leading to an increased risk for tumor hypermutation. However, the specific characteristics and clinical implications of TMZ-induced hypermutation remain poorly understood, and its relationship with immunotherapy has not been extensively investigated. This study presents a case report of a glioma patient who underwent 12 cycles of TMZ treatment and experienced hypermutation, while concurrently receiving adjuvant immunotherapy. Our findings suggest that TMZ-induced hypermutation may serve as a potential predictive marker for an unfavorable response to immunotherapy in cases of recurrent gliomas.

The role of TMZ therapy in patients with gliomas is well- established. Patients with LGG often have a younger age at diagnosis and a comparatively prolonged expected survival, leading to increased exposure to TMZ and a higher prevalence of hypermutation (10). TMZ-induced hypermutation is characterized by a significant increase in the mutation rate and a specific mutational signature involving G:C>A:T transitions during post-treatment recurrences (28). Previous studies have identified two main pathways to hypermutation: a *de novo* pathway associated with inherited defects in DNA polymerase and MMR genes, and a more common post-treatment pathway linked to acquired resistance driven by MMR defects in gliomas that recur after TMZ treatment (9).

However, the clinical relevance of TMZ-induced hypermutation remains uncertain. Bai et al. demonstrated that TMZ-induced hypermutation can drive malignant transformation in low-grade astrocytoma (29). In our study, the malignancy of the tumor also increased after TMZ treatment, as evidenced by higher tumor grade and Ki-67 index. Yu et al. confirmed that TMZ-induced hypermutation is associated with distant recurrence and reduced survival in low-grade IDH-mutant gliomas (11). Similarly, our case experienced subependymal dissemination (**Supplementary Figure 1**) and succumbed to the disease two months after hypermutation. Given the clinical significance of hypermutation, the factors that are predictive for risk of hypermutation should be specified. In our case, the time between initiation of TMZ treatment and tumor hypermutation was 27 months, and the duration of TMZ treatment comprised 12 cycles, consistent with previous findings (28). A prior study indicated that a high level of MGMT promoter methylation is a predictor of TMZ-induced hypermutation (30); however, this trend was not observed in the current report.

With regard to the clinical implication of hypermutation in guiding immunotherapy, it remains an important aspect that requires further investigation. Hypermutation, as an emerging biomarker, has been associated with a higher TMB, suggesting an increased tumor neoantigens and a potential for improved response to immunotherapy (18–20). Consequently, TMB has been utilized as an indicator to determine patient suitability for immunotherapy (31). However, research has revealed inconsistencies in the criteria for determining TMB, largely due to variations in detection methods. Furthermore, it is important to note that TMB alone does not directly correlate with the immunogenicity of tumor neoantigens, as it solely reflects antigen quantity while neglecting their quality (32). Notably, hypermutated gliomas exhibit a lack of prominent T cell infiltrates, extensive intratumoral heterogeneity, poor survival outcomes, and a low rate of response to immunotherapy (9). This suggests that hypermutation may not be sufficient for predicting an effective antitumor immune response (33).

In this particular case, sample A exhibited only 73 newly occurring mutations. The comprehensive immunophenoscore was 3, and the patient’s response to immunotherapy was moderate, without significant tumor remission. Conversely, sample B demonstrated a twofold increase in newly occurring mutations, accompanied by an immunophenoscore of 5. The patient displayed a favorable response to immunotherapy, with the tumor experiencing persistent partial remission. However, in sample C, the tumor underwent TMZ-induced hypermutation, resulting in a remarkable increase of 2466 newly identified mutations. Consequently, the comprehensive immunophenoscore significantly decreased, indicating a notable rise in intratumoral heterogeneity. Moreover, the tumor microenvironment exhibited an immune-suppressive state, and the subsequent patient outcome supported this observation. These findings are consistent with previous reports from Touat et al. (9), suggesting a potential association between TMZ-induced hypermutation and unfavorable response to immunotherapy. However, not all instances of hypermutations imply a compromised efficacy of immunotherapy. In the study conducted by Anghileri et al. in 2021, a patient with recurrent GBM and Lynch syndrome, characterized by a high tumor mutational burden, exhibited a strong immune response to anti-PD1 therapy (34). This response was attributed to a constitutional or biallelic MMR deficiency, resulting from germline alterations in the MMR gene. Such alterations induce a deficiency in the DNA repair machinery, leading to a hypermutable phenotype with an increased tumor mutational burden capable of generating neoantigens. The generation of a greater number of neoantigens correlates with improved clinical efficacy in immunotherapy. This phenomenon was also validated in another study that demonstrated the heightened sensitivity of hypermutated GBM patients with MMR deficiency to nivolumab treatment (35). In our case, no germline alterations were identified, yet specific mutations associated with MMR, including PMS2, MSH3, POLE, ATR and FANCA, were observed. Despite the absence of a definitive diagnosis of MMR deficiency based on sequencing results, these mutations were implicated as the primary contributors to hypermutation. Future verification of our speculation may entail conducting staining assays for MLH1, MSH2, MSH6, and PMS2. Thus, TMB cannot be solely relied upon as a standalone biomarker for forecasting the efficacy of immunotherapy in glioma patients (36, 37).

In summary, long-term administration of TMZ poses an increased risk of hypermutation in gliomas, leading to heightened tumor malignancy and the development of treatment resistance. TMZ-induced hypermutation may serve as an indicator associated with a negative response to immunotherapy in gliomas.

## Data availability statement

The original contributions presented in the study are included in the article/**Supplementary Materials**. Further inquiries can be directed to the corresponding authors.

## Ethics statement

The studies involving humans were approved by Institutional Review Board of Capital Medical University. The studies were conducted in accordance with the local legislation and institutional requirements. The participants provided their written informed consent to participate in this study. Written informed consent was obtained from the individual(s) for the publication of any potentially identifiable images or data included in this article.

## Author contributions

JL: Writing – review & editing, Formal Analysis, Methodology, Writing – original draft. SH: Writing – original draft, Writing – review & editing, Data curation, Resources. HJ: Resources, Writing – original draft, Writing – review & editing, Conceptualization, Formal Analysis, Funding acquisition, Supervision. YC: Conceptualization, Supervision, Writing – review & editing.

## Supplementary material

Supplementary Figure 1 Patient underwent subependymal dissemination recurrence after hypermutation.

## References

[1] Ostrom QT Price M Neff C Cioffi G Waite KA Kruchko C . Cbtrus statistical report: primary brain and other central nervous system tumors diagnosed in the United States in 2015-2019. Neuro Oncol. (2022) 24:v1–v95. doi: 10.1093/neuonc/noac202 36196752 PMC9533228

[2] Louis DN Perry A Wesseling P Brat DJ Cree IA Figarella-Branger D . The 2021 who classification of tumors of the central nervous system: A summary. Neuro Oncol. (2021) 23:1231–51. doi: 10.1093/neuonc/noab106 PMC8328013 34185076

[3] Jiang H Zhu Q Wang X Li M Shen S Yang C . Characterization and clinical implications of different Malignant transformation patterns in diffuse low-grade gliomas. Cancer Sci. (2023) 114:3708–18. doi: 10.1111/cas.15889 PMC10475770 37332121

[4] Sanai N Berger MS . Surgical oncology for gliomas: the state of the art. Nat Rev Clin Oncol. (2018) 15:112–25. doi: 10.1038/nrclinonc.2017.171 29158591

[5] van den Bent MJ Geurts M French PJ Smits M Capper D Bromberg JEC . Primary brain tumours in adults. Lancet. (2023) 402:1564–79. doi: 10.1016/S0140-6736(23)01054-1 37738997

[6] Stupp R Mason WP van den Bent MJ Weller M Fisher B Taphoorn MJ . Radiotherapy plus concomitant and adjuvant temozolomide for glioblastoma. N Engl J Med. (2005) 352:987–96. doi: 10.1056/NEJMoa043330 15758009

[7] Ostermann S Csajka C Buclin T Leyvraz S Lejeune F Decosterd LA . Plasma and cerebrospinal fluid population pharmacokinetics of temozolomide in Malignant glioma patients. Clin Cancer Res. (2004) 10:3728–36. doi: 10.1158/1078-0432.CCR-03-0807 15173079

[8] Tan AC Ashley DM Lopez GY Malinzak M Friedman HS Khasraw M . Management of glioblastoma: state of the art and future directions. CA Cancer J Clin. (2020) 70:299–312. doi: 10.3322/caac.21613 32478924

[9] Touat M Li YY Boynton AN Spurr LF Iorgulescu JB Bohrson CL . Mechanisms and therapeutic implications of hypermutation in gliomas. Nature. (2020) 580:517–23. doi: 10.1038/s41586-020-2209-9 PMC8235024 32322066

[10] Choi S Yu Y Grimmer MR Wahl M Chang SM Costello JF . Temozolomide-associated hypermutation in gliomas. Neuro Oncol. (2018) 20:1300–9. doi: 10.1093/neuonc/noy016 PMC6120358 29452419

[11] Yu Y Villanueva-Meyer J Grimmer MR Hilz S Solomon DA Choi S . Temozolomide-induced hypermutation is associated with distant recurrence and reduced survival after high-grade transformation of low-grade idh-mutant gliomas. Neuro Oncol. (2021) 23:1872–84. doi: 10.1093/neuonc/noab081 PMC8563321 33823014

[12] Geurts M van den Bent MJ . Hypermutated recurrences: identifying the clinical relevance. Neuro Oncol. (2021) 23:1805–6. doi: 10.1093/neuonc/noab192 PMC8563306 34455436

[13] Ngwa W Irabor OC Schoenfeld JD Hesser J Demaria S Formenti SC . Using immunotherapy to boost the abscopal effect. Nat Rev Cancer. (2018) 18:313–22. doi: 10.1038/nrc.2018.6 PMC5912991 29449659

[14] Zhang Z Liu X Chen D Yu J . Radiotherapy combined with immunotherapy: the dawn of cancer treatment. Signal Transduct Target Ther. (2022) 7:258. doi: 10.1038/s41392-022-01102-y 35906199 PMC9338328

[15] Wang H Xu T Huang Q Jin W Chen J . Immunotherapy for Malignant glioma: current status and future directions. Trends Pharmacol Sci. (2020) 41:123–38. doi: 10.1016/j.tips.2019.12.003 31973881

[16] Kreatsoulas D Bolyard C Wu BX Cam H Giglio P Li Z . Translational landscape of glioblastoma immunotherapy for physicians: guiding clinical practice with basic scientific evidence. J Hematol Oncol. (2022) 15:80. doi: 10.1186/s13045-022-01298-0 35690784 PMC9188021

[17] Sampson JH Gunn MD Fecci PE Ashley DM . Brain immunology and immunotherapy in brain tumours. Nat Rev Cancer. (2020) 20:12–25. doi: 10.1038/s41568-019-0224-7 31806885 PMC7327710

[18] Chan TA Yarchoan M Jaffee E Swanton C Quezada SA Stenzinger A . Development of tumor mutation burden as an immunotherapy biomarker: utility for the oncology clinic. Ann Oncol. (2019) 30:44–56. doi: 10.1093/annonc/mdy495 30395155 PMC6336005

[19] Zheng M . Tumor mutation burden for predicting immune checkpoint blockade response: the more, the better. J Immunother Cancer. (2022) 10:e003087. doi: 10.1136/jitc-2021-003087 35101940 PMC8804687

[20] Jardim DL Goodman A de Melo Gagliato D Kurzrock R . The challenges of tumor mutational burden as an immunotherapy biomarker. Cancer Cell. (2021) 39:154–73. doi: 10.1016/j.ccell.2020.10.001 PMC7878292 33125859

[21] Anagnostou V Bardelli A Chan TA Turajlic S . The status of tumor mutational burden and immunotherapy. Nat Cancer. (2022) 3:652–6. doi: 10.1038/s43018-022-00382-1 35764740

[22] Hypermutated gliomas respond poorly to immunotherapy. Cancer Discovery. (2020) 10:Of5. doi: 10.1158/2159-8290.CD-NB2020-045 32434948

[23] McGrail DJ Pilié PG Rashid NU Voorwerk L Slagter M Kok M . High tumor mutation burden fails to predict immune checkpoint blockade response across all cancer types. Ann Oncol. (2021) 32:661–72. doi: 10.1016/j.annonc.2021.02.006 PMC8053682 33736924

[24] Jiang H Yu K Cui Y Ren X Li M Yang C . Combination of immunotherapy and radiotherapy for recurrent Malignant gliomas: results from a prospective study. Front Immunol. (2021) 12:632547. doi: 10.3389/fimmu.2021.632547 34025640 PMC8138184

[25] Wang J Cazzato E Ladewig E Frattini V Rosenbloom DI Zairis S . Clonal evolution of glioblastoma under therapy. Nat Genet. (2016) 48:768–76. doi: 10.1038/ng.3590 PMC5627776 27270107

[26] Mutational Signatures (V3.4 - October 2023). Catalogue Of Somatic Mutations In Cancer (2023). Available at: https://cancer.sanger.ac.uk/signatures/.

[27] Charoentong P Finotello F Angelova M Mayer C Efremova M Rieder D . Pan-cancer immunogenomic analyses reveal genotype-immunophenotype relationships and predictors of response to checkpoint blockade. Cell Rep. (2017) 18:248–62. doi: 10.1016/j.celrep.2016.12.019 28052254

[28] Johnson BE Mazor T Hong C Barnes M Aihara K McLean CY . Mutational analysis reveals the origin and therapy-driven evolution of recurrent glioma. Science. (2014) 343:189–93. doi: 10.1126/science.1239947 PMC3998672 24336570

[29] Bai H Harmanci AS Erson-Omay EZ Li J Coskun S Simon M . Integrated genomic characterization of idh1-mutant glioma Malignant progression. Nat Genet. (2016) 48:59–66. doi: 10.1038/ng.3457 26618343 PMC4829945

[30] Mathur R Zhang Y Grimmer MR Hong C Zhang M Bollam S . Mgmt promoter methylation level in newly diagnosed low-grade glioma is a predictor of hypermutation at recurrence. Neuro Oncol. (2020) 22:1580–90. doi: 10.1093/neuonc/noaa059 PMC8444710 32166314

[31] Goodman AM Kato S Bazhenova L Patel SP Frampton GM Miller V . Tumor mutational burden as an independent predictor of response to immunotherapy in diverse cancers. Mol Cancer Ther. (2017) 16:2598–608. doi: 10.1158/1535-7163.Mct-17-0386 PMC5670009 28835386

[32] Rech AJ Balli D Mantero A Ishwaran H Nathanson KL Stanger BZ . Tumor immunity and survival as a function of alternative neopeptides in human cancer. Cancer Immunol Res. (2018) 6:276–87. doi: 10.1158/2326-6066.Cir-17-0559 PMC6047936 29339376

[33] Wang Q Hu B Hu X Kim H Squatrito M Scarpace L . Tumor evolution of glioma-intrinsic gene expression subtypes associates with immunological changes in the microenvironment. Cancer Cell. (2018) 33:152. doi: 10.1016/j.ccell.2017.12.012 29316430 PMC5892424

[34] Anghileri E Di Ianni N Paterra R Langella T Zhao J Eoli M . High tumor mutational burden and T-cell activation are associated with long-term response to anti-pd1 therapy in lynch syndrome recurrent glioblastoma patient. Cancer Immunol Immunother. (2021) 70:831–42. doi: 10.1007/s00262-020-02769-4 PMC10992921 33140187

[35] Bouffet E Larouche V Campbell BB Merico D de Borja R Aronson M . Immune checkpoint inhibition for hypermutant glioblastoma multiforme resulting from germline biallelic mismatch repair deficiency. J Clin Oncol. (2016) 34:2206–11. doi: 10.1200/jco.2016.66.6552 27001570

[36] Hodges TR Ott M Xiu J Gatalica Z Swensen J Zhou S . Mutational burden, immune checkpoint expression, and mismatch repair in glioma: implications for immune checkpoint immunotherapy. Neuro Oncol. (2017) 19:1047–57. doi: 10.1093/neuonc/nox026 PMC5570198 28371827

[37] Merchant M Ranjan A Pang Y Yu G Kim O Khan J . Tumor mutational burden and immunotherapy in gliomas. Trends Cancer. (2021) 7:1054–8. doi: 10.1016/j.trecan.2021.08.005 PMC10423405 34580037

