# Supplementary material for: Case report: Temozolomide induced hypermutation indicates an unfavorable response to immunotherapy in patient with gliomas
Source: Front Immunol. 2024 Apr 4;15:1369972. doi: 10.3389/fimmu.2024.1369972 (PMC11059094; doi:10.3389/fimmu.2024.1369972)
Supplement: Supplementary file 1 [file Table_1.docx]

Table S1. Enriched pathways for each cluster

| **Cluster** | **adj. P-value** | **nGenes** | **Pathways** |
| --- | --- | --- | --- |
| **A** | 6.9e-70 | 381 | Immune system process |
|  | 7.0e-68 | 316 | Immune response |
|  | 4.7e-57 | 260 | Defense response |
|  | 2.6e-51 | 284 | Response to external stimulus |
|  | 3.0e-51 | 200 | Immune effector process |
|  | 1.0e-45 | 193 | Leukocyte activation |
|  | 1.2e-45 | 206 | Cell activation |
|  | 1.3e-45 | 179 | Innate immune response |
|  | 1.5e-41 | 379 | Response to stress |
|  | 2.0e-41 | 221 | Regulation of immune system process |
|  | 6.5e-39 | 318 | Cellular response to chemical stimulus |
|  | 1.3e-37 | 260 | Positive regulation of response to stimulus |
|  | 4.0e-37 | 150 | Response to other organism |
|  | 8.1e-37 | 174 | Response to cytokine |
| **B** | 3.7e-15 | 106 | Nervous system development |
|  | 9.2e-13 | 76 | Generation of neurons |
|  | 2.1e-12 | 78 | Neurogenesis |
|  | 3.0e-12 | 38 | Regulation of trans-synaptic signaling |
|  | 4.7e-12 | 69 | Neuron differentiation |
|  | 1.4e-11 | 54 | Regulation of nervous system development |
|  | 3.3e-11 | 103 | Anatomical structure morphogenesis |
|  | 1.8e-10 | 45 | Anterograde trans-synaptic signaling |
|  | 1.8e-10 | 85 | Regulation of multicellular organismal development |
|  | 2.2e-10 | 75 | Cell-cell signaling |
|  | 6.6e-10 | 47 | Regulation of neurogenesis |
| **C** | 5.1e-22 | 31 | Nucleosome assembly |
|  | 6.4e-22 | 32 | Chromatin assembly |
|  | 3.6e-21 | 19 | Chromatin silencing at rDNA |
|  | 4.3e-20 | 17 | DNA replication-dependent nucleosome assembly |
|  | 1.1e-19 | 31 | Nucleosome organization |
|  | 6.0e-19 | 37 | DNA conformation change |
|  | 8.5e-18 | 33 | Protein-DNA complex assembly |
|  | 8.6e-17 | 23 | Chromatin silencing |
|  | 8.6e-17 | 25 | Protein heterooligomerization |
|  | 1.4e-16 | 22 | SRP-dependent cotranslational protein targeting to membrane |
|  | 4.6e-16 | 33 | Protein-DNA complex subunit organization |
| **D** | 4.0e-10 | 69 | Nervous system development |
|  | 1.2e-07 | 48 | Generation of neurons |
|  | 6.6e-07 | 43 | Neuron differentiation |
|  | 7.7e-06 | 36 | Neuron development |
|  | 8.4e-06 | 54 | Cell development |
|  | 1.9e-05 | 27 | Anterograde trans-synaptic signaling |
|  | 5.0e-05 | 30 | Regulation of nervous system development |
|  | 5.7e-05 | 44 | Cell-cell signaling |
|  | 1.1e-04 | 19 | Regulation of membrane potential |
|  | 1.2e-04 | 11 | Learning |
|  | 1.2e-04 | 30 | Neuron projection development |
