# Supplementary figures and images for: Case report: Temozolomide induced hypermutation indicates an unfavorable response to immunotherapy in patient with gliomas
Source: Front Immunol. 2024 Apr 4;15:1369972. doi: 10.3389/fimmu.2024.1369972 (PMC11059094; doi:10.3389/fimmu.2024.1369972)

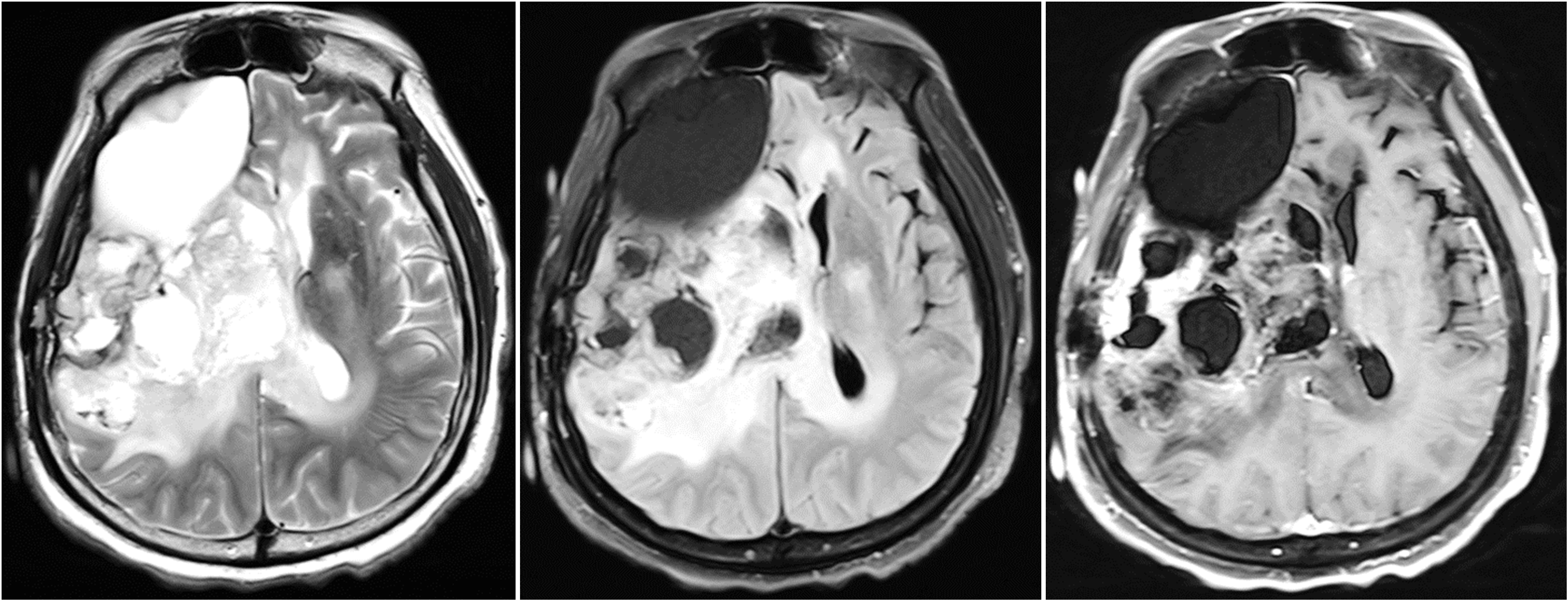

Supplement: Supplementary Figure 1 — Patient underwent subependymal dissemination recurrence after hypermutation. [file Image_1.jpeg]
